# Exploring the Ion Channel TRPV2 and Testicular Macrophages in Mouse Testis

**DOI:** 10.3390/ijms22094727

**Published:** 2021-04-29

**Authors:** Katja Eubler, Pia Rantakari, Heidi Gerke, Carola Herrmann, Annika Missel, Nina Schmid, Lena Walenta, Shibojyoti Lahiri, Axel Imhof, Leena Strauss, Matti Poutanen, Artur Mayerhofer

**Affiliations:** 1 Cell Biology-Anatomy III, Biomedical Center Munich (BMC), Faculty of Medicine, Ludwig-Maximilian-University (LMU), D-82152 Planegg-Martinsried, Germany; katja.eubler@bmc.med.lmu.de (K.E.); carola.herrmann@bmc.med.lmu.de (C.H.); annika.missel@bmc.med.lmu.de (A.M.); nina.schmid@bmc.med.lmu.de (N.S.); lena.walenta@gmail.com (L.W.); 2 Turku Bioscience Center, University of Turku and Åbo Akademi University, FI-20300 Turku, Finland; piaranta@utu.fi (P.R.); hmsulo@utu.fi (H.G.); 3 Protein Analysis Unit, Biomedical Center Munich (BMC), Faculty of Medicine, Ludwig-Maximilian-University (LMU), D-82152 Planegg-Martinsried, Germany; Shibojyoti.Lahiri@med.uni-muenchen.de (S.L.); aimhof@med.uni-muenchen.de (A.I.); 4 Research Center for Integrative Physiology and Pharmacology, Institute of Biomedicine, University of Turku, FI-20300 Turku, Finland; leesal@utu.fi (L.S.); matpou@utu.fi (M.P.); 5 Turku Center for Disease Modeling, Institute of Biomedicine, University of Turku, FI-20300 Turku, Finland

**Keywords:** TRPV2, testis, macrophages, aging, inflammation, AROM**^+^**, infertility

## Abstract

The cation channel TRPV2 is known to be expressed by murine macrophages and is crucially involved in their functionality. Macrophages are frequent cells of the mouse testis, an immune-privileged and steroid-producing organ. TRPV2 expression by testicular macrophages and possible changes associated with age or inflammation have not been investigated yet. Therefore, we studied testes of young adult and old wild-type (WT) and AROM**^+^** mice, i.e., transgenic mice overexpressing aromatase. In these animals, inflammatory changes are described in the testis, involving active macrophages, which increase with age. This is associated with impaired spermatogenesis and therefore AROM**^+^** mice are a model for male infertility associated with sterile inflammation. In WT animals, testicular TRPV2 expression was mapped to interstitial CD206**^+^** and peritubular MHC II**^+^** macrophages, with higher levels in CD206**^+^** cells. Expression levels of TRPV2 and most macrophage markers did not increase significantly in old mice, with the exception of CD206. As the number of TRPV2**^+^** testicular macrophages was relatively small, their possible involvement in testicular functions and in aging in WT mice remains to be further studied. In AROM**^+^** testis, TRPV2 was readily detected and levels increased significantly with age, together with macrophage markers and TNF-α. TRPV2 co-localized with F4/80 in macrophages and further studies showed that TRPV2 is mainly expressed by unusual CD206**^+^**MHC II**^+^** macrophages, arising in the testis of these animals. Rescue experiments (aromatase inhibitor treatment and crossing with ERαKO mice) restored the testicular phenotype and also abolished the elevated expression of TRPV2, macrophage and inflammation markers. This suggests that TRPV2^+^ macrophages of the testis are part of an inflammatory cascade initiated by an altered sex hormone balance in AROM**^+^** mice. The changes in testis are distinct from the described alterations in other organs of AROM^+^, such as prostate and spleen. When we monitored TRPV2 levels in another immune-privileged organ, namely the brain, we found that levels of TRPV2 were not elevated in AROM^+^ and remained stable during aging. In the adrenal, which similar to the testis produces steroids, we found slight, albeit not significant increases in TRPV2 in both AROM^+^ and WT mice, which were associated with age. Thus, the changes in the testis are specific for this organ.

## 1. Introduction

Transgenic mice overexpressing aromatase (AROM^+^) have been studied for many years [1,2,3]. The hormonal imbalance in these animals causes immunological changes and, in some organs, inflammation. A recent paper described higher plasma immunoglobulin levels, mainly IgE, in AROM^+^ mice and distinct changes in the enlarged spleen [4]. The analysis of splenocytes revealed changes in the ratio of mature/immature B cells and increased IgE synthesis after IgE class-switching. Inflammatory changes in two organs were described, namely in the prostate and testis. In prostate, inflammation (prostatitis) and pre-malignant changes were found and occurred in an age-dependent manner [5]. In the inflammatory lesions several immune cell types, including mast cells, neutrophils, T-lymphocytes, and F4/80^+^ macrophages were present. In testis of AROM^+^, the most striking observations were the age-dependent increase in testicular macrophages positive for CD68, elevated TNF-α levels, and the parallel decline in spermatogenesis [1,2,6,7]. The changes became detectable at about 2 months of age and further increased with age. Of note, in many cases of human idiopathic male infertility due to impaired spermatogenesis, massive increases in testicular macrophage numbers, defined by the expression of CD68, occur, as well [8]. This and other similar changes led to the assumption that AROM^+^ mice are a model for human male infertility associated with sterile inflammation [9].

Tissue-resident macrophages of the testis have mainly been studied in rodents, and these studies indicated that macrophages normally participate in a broad spectrum of testis-specific functions, i.e., regulation of vascularization and morphogenesis [10], support of Leydig cell function [11], differentiation of spermatogonia [12], and maintenance of the immune privilege [13,14]. Macrophages are typically classified into inflammatory-activated (M1) and alternatively activated immunosuppressive macrophages (M2). M1 macrophages secrete proinflammatory mediators including large amounts of the proinflammatory cytokine TNF-α. M2 macrophages are essential for tissue homeostasis, immune surveillance, and inflammation resolution and secrete low amounts of TNF-α [15]. For rodent testicular macrophages a polarization towards the immunoregulatory and immunotolerant M2 phenotype is typical. However, it is becoming clear that this state is not fixed and that the local environment governs the polarization of testicular macrophages [16]. Such regulatory influences play a role in inflammation and possibly also in aging of the testis. The last-mentioned point is not well known, but changes in testicular macrophage numbers during aging have been reported in rodents [17].

From a developmental point of view, the murine testis normally contains two different populations of macrophages, which have their origin predominantly in the fetal liver, as just very recently demonstrated [18]. Already at birth, a testicular compartment, i.e., the interstitial space is occupied by CD206**^+^**MHC II**^-^** macrophages, whereas CD206**^-^**MHC II**^+^** ones surround the seminiferous tubules. Furthermore, a new study showed that after radiation or during infection, bone marrow-derived circulating monocytes are recruited to the testis and give rise to inflammatory macrophages which then promote tissue damage [19]. Hence, testicular macrophages can be heterogeneous.

The family of transient receptor potential (TRP) channels consists of a large group of rather ubiquitously expressed ion channels with a broad spectrum of functions and activating stimuli and is further divided into nine subfamilies (TRPA, TRPC, TRPM, TRPML, TRPN, TRPP, TRPS, TRPV, TRPVL), each comprising members with structural homology. All these channels have in common a relatively non-selective permeability for cations and all are regarded to act as molecular sensors. Among these channels, the transient receptor potential channel subfamily V member 2 (TRPV2) remains one of the least known channels [20]. It was considered as a thermal sensor for noxious heat > 50 °C [21] for a long time, however, TRPV2 has a much more complex physiology and properties, as demonstrated by knock-out mice [22]. Furthermore, the properties strongly depend on the species and cell type [23]. The known modulators include, in addition to noxious heat, Δ9-tetrahydrocannabinol (THC), cannabidiol (CBD), and 2-amino-ethoxydiphenyl borat (2-APB), acting as activators [24,25], and tranilast and ruthenium red, which represent antagonists [26,27], respectively. Yet all of these modulators lack specificity for TRPV2. Only very recently, it could be demonstrated that oxidation of a methionine residue within the structure of TRPV2 by reactive oxygen species (ROS), such as H_2_O_2_, or ultraviolet A light leads to activation and sensitization of TRPV2 [28], giving rise to endogenous regulatory mechanisms.

TRPV2 is reported to be expressed in different organs, including the brain [29], pancreas [30], muscle [31], spleen [21], and other immune-related tissues [32]. In the last decades, TRPV2 has been described to be crucially involved in innate and adaptive immunity and has been found in several cell types of the immune system in both human and mouse, i.e., in macrophages and osteoclasts, mast cells, granulocytes, monocytes, and also in T-lymphocytes [33,34,35,36]. In most of these cells, TRPV2 is essential for migration and it appears to be irreplaceable for differentiation of osteoclasts [37] or degranulation of mast cells [33,36].

In macrophages, TRPV2 is essential for phagocytosis and chemotaxis [34,35,38]. TRPV2-deficient mice exhibit a higher bacterial load and increased mortality [22]. TRPV2 expression in macrophages was mainly demonstrated for resident populations, e.g., of brain, liver, skin, lung, and bone [39,40]. While a sizable number of organs and their respective macrophages were analyzed, the testis and in particular testicular macrophages have not been studied.

This paucity of information led us to perform this study. We examined, whether TRPV2 is expressed by mouse testicular macrophages, attempted to define the macrophage subtype by flow cytometry and studied, whether levels may change in old animals. Since TRPV2 is associated with inflammation we specifically focused on the testis of AROM**^+^** mice [1,2]. Additionally, the testicular tissue from known successful rescue experiments, i.e., treatment with an aromatase inhibitor [6] and crossbreeding with ERαKO animals [41,42], were included in this study to gain insights into the regulation of TRPV2. Finally, we explored the expression of *TRPV2* in the murine adrenal, i.e., an organ of steroid production, similar to the testis, and *TRPV2* levels in the brain, which is similar to the testis, an immune-privileged organ.

## 2. Results

### 2.1. Testicular Expression of TRPV2, Macrophage and Inflammation Markers in Young and Old WT Mice

Whole testes lysates of young (2–3 months, *n* = 21) and old WT mice (6–8 months, *n* = 9) were subjected to a detailed analysis employing quantitative RT-PCR and Western blotting. Next to TRPV2 and *NOX2*, we examined macrophage and inflammation markers. Testicular sections were further investigated by means of TRPV2 in situ hybridization and immunohistochemistry. In addition, we incubated the testicular tissue with cannabidiol (CBD), known to activate TRPV2, and screened for consequences of activation of TRPV2.

**Figure 1:**
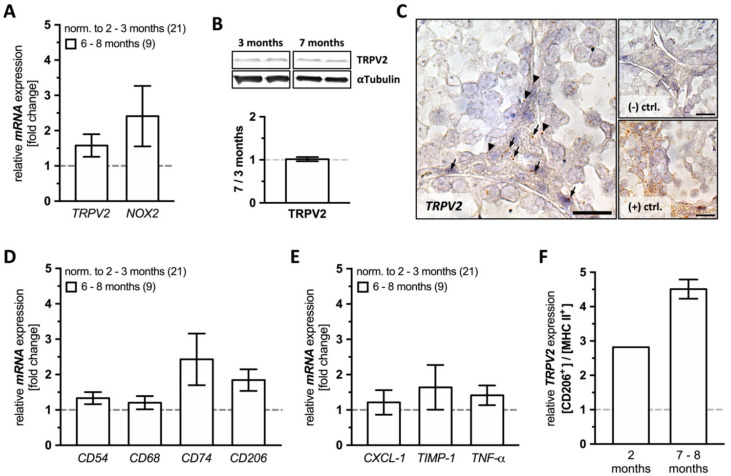
Expression profile of *TRPV2*, *NOX2*, macrophage and inflammation markers in WT testes. (**A**) Relative mRNA expression levels of *TRPV2* and *NOX2* in whole testes of old WT mice, normalized to young animals. Levels were not significantly different (*TRPV2*: 1.578 ± 0.319, *p* = 0.3505; *NOX2*: 2.412 ± 0.856, *p* = 0.2802). (**B**) TRPV2 immunoblotting of young (3 months, *n* = 2) and old WT testes (7 months, *n* = 2) showed weak bands (~63 kDa) for both ages and quantification revealed a mean expression level in old WT testis of 1.016 ± 0.049, compared to young ones. Staining for αTubulin was used as a loading control. (**C**) Testicular sections of 10 month-old WT mice subjected to *TRPV2* in situ hybridization showed punctuated staining mainly in the interstitial space (arrows) with few spots at the peritubular walls (arrow heads; left panel). The negative control (upper right panel, (−) ctrl.) showed no signals, but the positive control (lower right panel, (+) ctrl.) did. Scale bar is 50 µM. (**D**,**E**) Relative mRNA expression levels of (**D**) macrophage markers (*CD54*: 1.332 ± 0.172, *p* = 0.1157; *CD68*: 1.205 ± 0.188, *p* = 0.8506; *CD74*: 2.430 ± 0.732, *p* = 0.2402; *CD206*: 1.843 ± 0.305, *p* = 0.0589) and (**E**) inflammation markers (*CXCL-1*: 1.214 ± 0.348, *p* = 0.6767; *TIMP-1*: 1.638 ± 0.633, *p* = 0.7160; *TNF-α*: 1.416 ± 0.279, *p* = 0.5198) in whole testes of old WT mice normalized to young animals. (**A**,**D**,**E**) The graphs represent mean ± SEM of old WT mice (6–8 months, *n* = 9) normalized to young animals (2–3 months, *n* = 21); unpaired two-tailed *t*-test, α = 0.05. (**F**) Testicular macrophages from young (2 months, *n* = 1, pool of three animals) and old WT mice (7–8 months, *n* = 2, pool of three animals each) sorted for their CD206 and MHC II expression by FACS were subjected to mRNA extraction and quantitative RT-PCR for *TRPV2* expression. The graphs represent mean ± SEM.

*TRPV2* was detected at the mRNA level in young and old mice, and a slight, although not statistically significant increase of *TRPV2* levels was found in old animals (Figure 1A). Immunoblotting did not reveal changes in TRPV2 level with increasing age (Figure 1B). Levels of the ROS generating enzyme *NOX2* were slightly but not significantly higher in older animals (Figure 1A).

When testicular sections of both young (2 months, Figure S1A) and even older, 10 month-old, WT animals were examined by *TRPV2* in situ hybridization (Figure 1C), we observed a distinct localization in the interstitial space (arrows) and within or in close proximity to the peritubular wall of seminiferous tubules (arrow heads).

Based on a detailed quantitative RT-PCR analysis, levels of all investigated macrophage markers, i.e., *CD54*, *CD68*, *CD74,* and *CD206*, remained unchanged in old WT animals (Figure 1D), as well as the examined markers for inflammation, i.e., *CXCL-1*, *TIMP-1,* and *TNF-α* (Figure 1E).

To further characterize the macrophage subpopulation(s) expressing *TRPV2*, testicular macrophages were isolated and subjected to FACS for expression of CD206 and MHC II and then scanned for *TRPV2* expression by quantitative RT-PCR (Figure 1F). In both young and old WT animals, CD206**^+^** macrophages showed numerically higher *TRPV2* expression levels compared to MHC II**^+^** ones (young: 2.820; old: 4.510 ± 0.280). This result suggests that predominantly interstitial macrophages express *TRPV2*, at least at the mRNA level (Figure 1C,F).

Since there was no change in TRPV2 expression in older animals, consequences of TRPV2 activation were explored in an organotypic incubation with testicular tissue from young WT mice (2–3 months, *n* = 6). The testicular tissue was pre-incubated with cannabinoid receptor 1 (CB1) and 2 (CB2) blockers, AM351 (80 nM) and AM630 (800 nM), respectively, for 1 h and then exposed to CBD (30 µM) for 6 h.

Using a proteome profiler, a 2.596-fold higher CXCL-1 level could be detected in the supernatant of CBD treated tissue (4031.422 signal density/µg RNA) compared to the control tissue (1553.138 signal density/µg RNA) and an albeit weak signal for IFN-y was found exclusively in the CBD-treated tissue supernatant (128.919 signal density/µg RNA; Figure S1B, upper panel).

A detailed quantitative RT-PCR analysis performed with mRNA isolated from the individual tissue pieces revealed significantly higher levels of *CXCL-1* (1.767 ± 0.326, *p* = 0.0493), *CXCL-2* (1.807 ± 0.334, *p* = 0.0180), and *IL-6* (1.697 ± 0.174, *p* = 0.0066) upon exposure to 30 µM CBD, whereas *CD54*, *COX2*, *IL-1ß*, *MCP-1*, *TIMP-1*, *TNF-α,* and *TRPV2* remained unchanged (Figure S1B, lower panel).

### 2.2. TRPV2, Macrophage and Inflammation Markers in Testes from Young and Old AROM^+^ Mice

**Figure 2:**
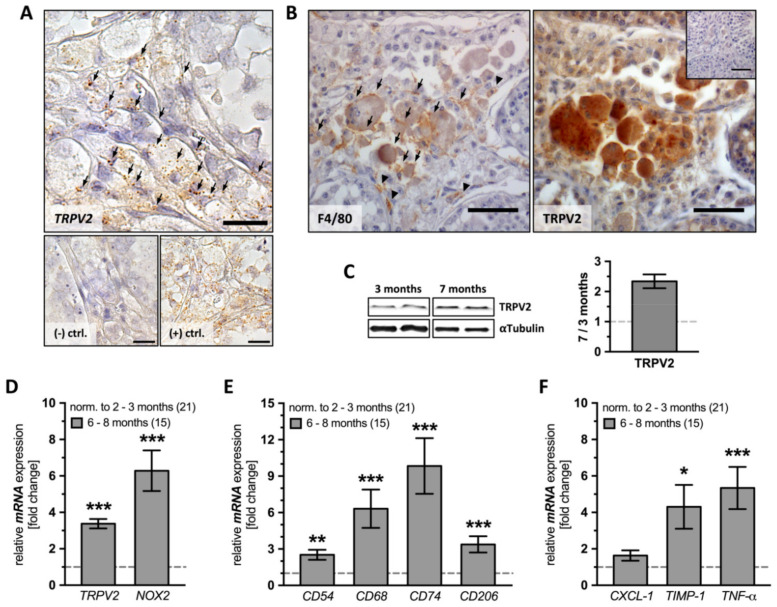
Expression profile of TRPV2, *NOX2*, macrophage and inflammation markers in AROM^+^ testes. (**A**) Testicular sections of 10 month-old AROM**^+^** mice subjected to *TRPV2* in situ hybridization (upper panel) revealed strong and punctuated staining mainly in the interstitial space (arrows). The negative control (lower left panel, (−) ctrl.) showed no signals, but the positive control (lower right panel, (+) ctrl.) did. Scale bar is 50 µm. (**B**) Consecutive testicular sections of 10 month-old AROM**^+^** animals subjected to immunohistochemistry using antibodies against the general murine macrophage marker F4/80 (left panel) and TRPV2 (right panel) revealed strong membrane-associated F4/80 staining in normal-sized and hyperplastic cells in the interstitial space (arrows), with some faint staining also within or close to the peritubular wall (arrow heads). TRPV2 showed a strong membrane-associated signal in the interstitial space, in both normal-sized and hyperplastic cells, whereas peritubular walls remained clear of any signal. The insert represents a negative control with the 1^st^ antibody omitted. Scale bar is 50 µm. (**C**) TRPV2 immunoblotting of whole testis lysates of young (3 months, *n* = 2) and old AROM**^+^** animals (7 months, *n* = 2) showed that bands at the expected size and quantification confirmed increased expression levels on the protein level. αTubulin was used as a loading control. (**D**–**F**) The mRNA of whole testis lysates from young and old AROM**^+^** mice was subjected to quantitative RT-PCR to investigate the expression profile of (**D**) *TRPV2* and *NOX2*, (**E**) macrophage and (**F**) inflammation markers revealing highly significant differences except for the inflammation marker *CXCL-1* (1.631 ± 0.286, *p* = 0.1034). The graphs represent the mean ± SEM of old AROM**^+^** mice (6–8 months, *n* = 15) normalized to young animals (2–3 months, *n* = 21); unpaired two-tailed *t*-test, α = 0.05; * *p* ≤ 0.05, ** *p* ≤ 0.01, *** *p* ≤ 0.001.

In the testes of AROM**^+^** animals, TRPV2 was readily detected already in young adults, as shown by several methods. Testicular sections of both young (2 months, Figure S2) and old AROM**^+^** mice (10 months) were subjected to *TRPV2* in situ hybridization. The results showed strong punctuated staining in the interstitial space, indicating that TRPV2 is present in large cells (Figure 2A). Immunohistochemistry using antibodies against the murine macrophage marker F4/80 (left panel) and TRPV2 (right panel) was also performed on consecutive sections from old AROM**^+^** animals (10 months). Immunoreactive signals indicate the co-localization of the two proteins and support the fact that testicular macrophages express TRPV2 in AROM**^+^** mice (Figure 2B).

Protein extracted from whole testis lysates of both young (3 months, *n* = 2) and old AROM**^+^** animals (7 months, *n* = 2) further confirmed the increase in TRPV2 amount (1.987 ± 0.378; Figure 2C).

Comparing young (2–3 months, *n* = 21) and old AROM**^+^** mice testes (6–8 months, *n* = 15) by means of quantitative RT-PCR showed that levels of both *TRPV2* (3.375 ± 0.257, *p* ≤ 0.0001) and *NOX2* (6.283 ± 1.114, *p* ≤ 0.0001) presented highly significant increases with age (Figure 2D).

Moreover, we found a highly significant increase for all investigated macrophage markers (*CD54*: 2.521 ± 0.415, *p* = 0.0010; *CD68*: 6.318 ± 1.580, *p* ≤ 0.0001; *CD74*: 9.838 ± 2.292, *p* ≤ 0.0001; *CD206*: 3.377 ± 0.669, *p* = 0.0002; Figure 2E), and, as expected, also *TNF-α* (5.341 ± 1.158, *p* ≤ 0.0001) and *TIMP-1* (4.307 ± 1.205, *p* = 0.0292) were significantly increased. However, CXCL-1 was not changed (Figure 2F).

### 2.3. TRPV2, Macrophage and Inflammation Markers in a Genotypic Comparison

**Figure 3:**
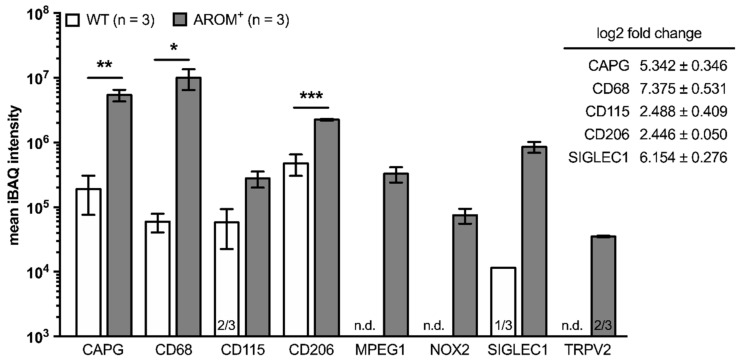
Mass spectrometry-based comparison of expression profiles in old WT and AROM^+^ testes. Extracted proteins from testicular tissue of 11 month-old WT and AROM**^+^** animals (*n* = 3, each) were subjected to mass spectrometry. The graph presents iBAQ intensities (mean ± SEM) of detected proteins and results of statistical analysis of a genotype-dependent comparison when proteins could be detected in all replicates using an unpaired two-tailed *t*-test with α = 0.05; * *p* ≤ 0.05, ** *p* *≤* 0.01, *** *p* ≤ 0.001. The numbers of positive replicates other than 3/3 are depicted at the bottom of each column (n.d.: not detected). The insert on the right depicts the mean differential expression of detected proteins (log2 fold change) in testes from AROM^+^ animals compared to coeval WT animals, if calculable.

For a better appreciation of the differences between the observed findings in testes from young and old WT and AROM**^+^** mice, on the one hand, quantitative RT-PCR datasets were re-analyzed comparing coeval animals of both genotypes (Figure S3) and on the other hand, datasets of proteomes from both WT and AROM**^+^** testes of 11 month-old animals subjected to mass spectrometry were re-analyzed and screened for macrophage and inflammation markers, TRPV2 and ROS generating enzymes (Figure 3).

In testes from young AROM**^+^** animals, *TRPV2* mRNA expression levels were found to be significantly higher compared to coeval WT mice (2.464 ± 0.365, *p* = 0.0029), and the difference between the two genotypes was further strongly augmented with the increasing age (5.217 ± 0.397, *p* ≤ 0.0001). In terms of testicular *NOX2*, there were significantly higher levels of the ROS producing enzyme in AROM^+^ mice compared to coeval WT mice (Figure S3A), both in young (3.049 ± 0.602, *p* = 0.0210) and old animals (8.604 ± 1.526, *p* ≤ 0.0001).

Comparison of macrophage marker expression levels in testes from young WT and AROM**^+^** animals revealed significantly higher levels of *CD54* (2.772 ± 0.431, *p* ≤ 0.0001) and *CD68* (3.441 ± 0.304, *p* ≤ 0.0001), but not of *CD74* and *CD206*. However, in old animals all analyzed macrophage markers showed significantly higher expression levels in AROM**^+^** animals compared to coeval WT mice (*CD54*: 4.062 ± 0.539, *p* ≤ 0.0001; *CD68*: 18.610 ± 4.654, *p* ≤ 0.0001; *CD74*: 6.488 ± 1.511, *p* = 0.0022; *CD206*: 2.399 ± 0.475, *p* = 0.0176; Figure S3B).

In terms of inflammation markers, already young AROM**^+^** animals featured significantly higher *CXCL-1* (2.649 ± 0.319, *p* = 0.0012), *TIMP-1* (4.787 ± 0.502, *p* ≤ 0.0001) and *TNF-α* expression levels (2.518 ± 0.375, *p* = 0.0057) compared to coeval WT mice. An observation being even stronger in the old mice of the two genotypes (*CXCL-1*: 5.071 ± 0.889, *p* = 0.0007; *TIMP-1*: 22.990 ± 6.436, *p* = 0.0002; *TNF-α*: 9.102 ± 1.973, *p* ≤ 0.0001; Figure S3C), indicates the increasing severity of the inflammatory testicular phenotype with age.

In a recent study [3], proteomes from both WT and AROM**^+^** testes of 11 month-old animals (*n* = 3, each) were studied by mass spectrometry and the datasets were screened for macrophage markers, inflammation markers, TRPV2 and ROS generating enzymes (Figure 3). The macrophage markers CAPG, CD68, and CD206 were readily found in all three animals of both genotypes with the mean iBAQ intensities being significantly higher in testes from AROM animals (CAPG: *p* = 0.0097; CD68: *p* = 0.0484; CD206: *p* = 0.0007). In addition, CD115, MPEG1, and SIGLEC1 could be detected in all three analyzed AROM^+^ mice, while in the WT animals CD115 was only present in two out of three animals, SIGLEC1 only in one and MPEG1 was even exclusively found in AROM^+^ mice.

Furthermore, next to MPEG1, both TRPV2 and NOX2, being the only ROS generating enzyme detected within these datasets, were exclusively found in AROM**^+^** animals by this experimental approach.

### 2.4. Flow Cytometry Analysis of Testicular Macrophages from Young WT and AROM^+^ Mice

**Figure 4:**
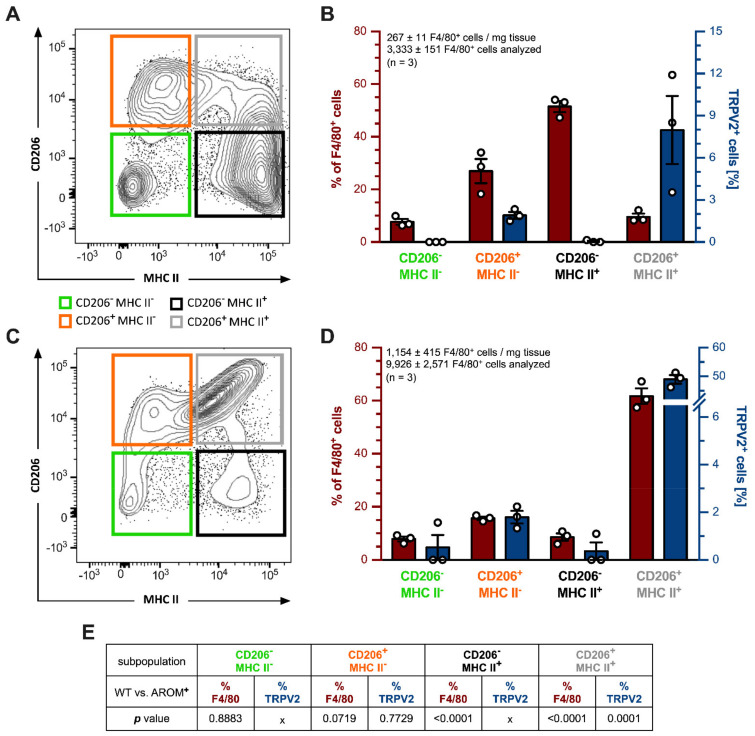
Flow cytometry analysis of testicular macrophage populations of young WT and AROM^+^ mice. Cells from 3 month-old WT and AROM**^+^** testes (*n* = 3, each) were isolated, processed for FACS analysis and sorted for macrophage markers (CD45**^+^**CD11b**^+^**F4/80**^+^**), and further divided by CD206 and MHC II expression. TRPV2 expression was quantified within the resulting subpopulations. (**A**,**C**) Representative two-dimensional plots of a WT (**A**) and AROM**^+^** animal (**C**), showing the four subpopulations depending on their CD206 and MHC II expression level (green—CD206**^-^**MHC II**^-^**, orange—CD206**^+^**MHC II**^-^**, black—CD206**^-^**MHC II**^+^**, gray—CD206**^+^**MHC II**^+^**). (**B**,**D**) Quantitative analyses of testicular macrophage subpopulations in WT and AROM**^+^** mice. The percentages of total F4/80**^+^** cells (red axis), and TRPV2**^+^** cells within these particular subpopulations (blue axis) are shown. The graphs represent mean ± SEM. (**E**) Results of statistical analysis (*p* values) of the particular subpopulations (% of F4/80^+^) and the corresponding percentage of TRPV2^+^ cells (TRPV2^+^ cells [%]) within these subpopulations of WT and AROM^+^ animals, using an unpaired two-tailed *t*-test, α = 0.05, where Gaussian distribution is given and proteins could be detected in all replicates (3/3).

To obtain an in-depth insight into the true nature of the testicular macrophages being positive for TRPV2, whole testes of young WT and AROM**^+^** mice (3 months, *n* = 3 each) were digested, and isolated cells were subjected to the flow cytometry (FACS) analysis. CD45**^+^**CD11b**^+^**F4/80**^+^** macrophages were further subdivided into four subpopulations based on their CD206 and MHC II expression (CD206**^-^**MHC II**^-^**, CD206**^+^**MHC II**^-^**, CD206**^-^**MHC II**^+^**, CD206**^+^**MHC II**^+^**) and sorted (Figure 4A,C).

Testes from WT animals (*n* = 3) harbored a total of 267 ± 11 CD45**^+^**CD11b**^+^**F4/80**^+^** cells per mg tissue and 3333 ± 151 of these cells were subjected to the flow cytometry analysis. The biggest fraction of these cells could be allotted to CD206**^-^**MHC II**^+^** macrophages (51.470 ± 2.099%), followed by CD206**^+^**MHC II**^-^** (26.970 ± 4.605%), CD206**^+^**MHC II**^+^** (9.583 ± 1.260%), and CD206**^-^**MHC II**^-^** ones (7.677 ± 1.107%; Figure 4B, plotted on the left red axis). Amongst these macrophage subpopulations, the double-positive group of CD206**^+^**MHC II**^+^** macrophages had the highest percentage of TRPV2**^+^** cells with 7.893 ± 2.424% (3/3). CD206**^+^**MHC II**^-^** macrophages harbored 1.903 ± 0.241% TRPV2**^+^** cells (3/3), whereas only in one animal (1/3) 0.140% of CD206**^-^**MHC II**^+^** macrophages were also positive for TRPV2, giving further support to the findings made on mRNA expression levels from sorted macrophages (Figure 1F). The double negative CD206**^-^**MHC II**^-^** subpopulation was bare of any TRPV2**^+^** cells (0/0) (Figure 4B, plotted on the right blue axis).

In contrast, testes from AROM**^+^** animals (*n* = 3) contained substantially more CD45**^+^**CD11b**^+^**F4/80**^+^** cells with 1154 ± 415 cells per mg tissue and 9926 ± 2571 cells were subjected to the flow cytometry analysis revealing a drastically shifted distribution of macrophage subpopulations towards the double-positive CD206**^+^**MHC II**^+^** subpopulation with 61.730 ± 2.924% of all F4/80**^+^** cells (Figure 4C), and 48.970 ± 1.488% of those being also TRPV2**^+^** (3/3). The double negative CD206**^-^**MHC II**^-^** subpopulation represented only 7.890 ± 0.900% of all F4/80**^+^** cells with 1.570% being also TRPV2**^+^** (1/3). CD206**^+^**MHC II**^-^** macrophages summed up to 15.670 ± 0.639% and CD206**^-^**MHC II**^+^** subpopulations came to 8.593 ± 1.375% of total F4/80**^+^** cells. Moreover, applying also for AROM**^+^** mice, CD206**^+^**MHC II**^-^** macrophages showed more TRPV2**^+^** cells with 1.793 ± 0.263% (3/3) than those macrophages being CD206**^-^**MHC II**^+^** with only 1.100% (1/3), respectively (Figure 4D).

For a comparison between the results obtained from these CD45**^+^**CD11b**^+^**F4/80**^+^** cells derived from WT and AROM^+^ mice, datasets were statistically analyzed using a two-tailed unpaired *t*-test where Gaussian distribution was given and the corresponding protein could be detected in all three replicates of each group (Figure 4E). In terms of macrophage subpopulation composition (% of F4/80^+^ cells), there was no significant difference in the amount of CD206**^-^**MHC II**^-^** (*p* = 0.8883) and CD206**^+^**MHC II**^-^** (*p* = 0.0719) cells between the two genotypes. However, in AROM**^+^** animals there were significantly fewer CD206**^-^**MHC II**^+^** macrophages (*p* ≤ 0.0001), but the percentage of the double-positive subpopulation of CD206**^+^**MHC II**^+^** cells was significantly higher in these mice (*p* ≤ 0.0001).

Expression of TRPV2 within these macrophage subpopulations (TRPV2^+^ cells [%]) could only be statistically investigated for CD206**^+^**MHC II^-^ and CD206**^+^**MHC II**^+^** cells, since TRPV2 could be found in all the animals examined. For CD206**^+^**MHC II**^-^** macrophages, there was no significant difference in TRPV2^+^ cells between the two genotypes (*p* = 0.7729), while in AROM**^+^** animals the double-positive CD206**^+^**MHC II**^+^** cells included significantly more TRPV2**^+^** macrophages (*p* = 0.0001) compared to coeval WT mice.

### 2.5. TRPV2 in Rescue Experiments of AROM**^+^** Mice

In previous studies [6,42], the altered endocrine phenotype of AROM**^+^** testis was rescued by administration of an aromatase inhibitor or by cross-breeding with estrogen receptor α knock-out (ERαKO) animals. We examined whether under these conditions elevated TRPV2 and *NOX2* but also macrophage and inflammation markers are reverted. 

Briefly, 1 month-old WT and AROM^+^ animals were exposed to an aromatase inhibitor (finrozole) or corresponding placebo control (carboxylmethyl-cellulose) for 6 weeks and mRNA was isolated from whole testis lysates after sacrifice of the animals for further analysis.

The data revealed that administration of the aromatase inhibitor caused only slight, but partially statistically significant changes in WT animals (*n* = 7), i.e., a slight increase in *CD206* mRNA expression (1.825 ± 0.183, *p* = 0.0153), whereas none of the other investigated markers showed significant changes compared to the placebo-treated WT animals (*n* = 7).

The analysis of placebo-treated AROM**^+^** animals (*n* = 7) revealed expected results with significantly increased expression levels of *TRPV2* and *NOX2*, as well as the majority of the macrophage and inflammation markers (*TRPV2*: 1.692 ± 0.094, *p* = 0.0369; *NOX2*: 3.094 ± 0.532, *p* = 0.0078; *CD54*: 3.940 ± 0.602, *p* = 0.0211; *CD68*: 2.562 ± 0.244, *p* ≤ 0.0001; *CD206*: 2.113 ± 0.219, *p* = 0.0060; *TIMP-1*: 1.722 ± 0.150, *p* = 0.0004; *TNF-α*: 4.026 ± 0.841, *p* = 0.0014). However, *CD74* (0.533 ± 0.069, *p* = 0.0323) was significantly decreased and *CXCL-1* was indistinguishable from the placebo-treated WT animals (2.185 ± 0.333, *p* = 0.1204;).

**Figure 5:**
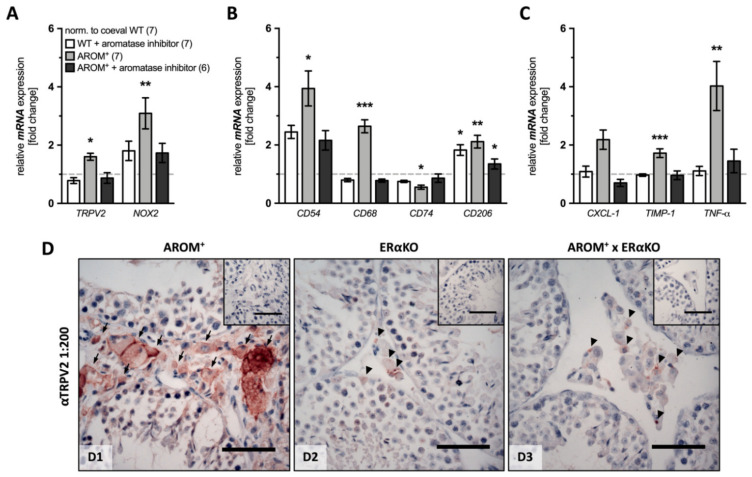
Rescue experiments of AROM^+^ mice prevent TRPV2 increases. (**A**–**C**) One month-old WT and AROM^+^ mice were treated with an aromatase inhibitor or with corresponding placebo for 6 weeks and whole testes were analyzed for their mRNA expression profile of (**A**) *TRPV2* and *NOX2*, (**B**) macrophage and (**C**) inflammation markers. The graphs represent mean ± SEM of inhibitor-treated WT (*n* = 7, white bars) and AROM^+^ mice treated with either placebo (*n* = 7, light grey bars) or aromatase inhibitor (*n* = 6, dark grey bars) compared to coeval placebo-treated WT animals (*n* = 7); unpaired two-tailed *t*-test, α = 0.05; * *p* ≤ 0.05, ** *p* ≤ 0.01, *** *p* ≤ 0.001. (**D**) Immunohistochemistry for TRPV2 performed on testicular sections from 9 month-old AROM^+^ (**D1**), ERαKO (**D2**) and AROM^+^ mice crossbred with ERαKO mice (**D3**) revealed strong positive signals in the interstitial space of AROM^+^ mice mainly membranous in normal-sized and hyperplastic cells (arrows), but only very weak and faint staining within the interstitial space of ERαKO and in AROM^+^ mice crossbred with ERαKO animals, with a rather punctuated pattern (arrow heads). The inserts depict corresponding controls with the omitted 1^st^ antibody. Scale bar is 50 µm.

The inhibitor treatment had a strong impact on AROM**^+^** animals (*n* = 6) and, except for *CD206* (1.350 ± 0.172, *p* = 0.0491), levels of all markers were significantly indistinguishable from the ones of placebo-treated WT (*TRPV2*: 0.867 ± 0.181, *p* = 0.3956; *NOX2*: 1.731 ± 0.329, *p* = 0.2692; *CD54*: 2.160 ± 0.332, *p* = 0.1934; *CD68*: 0.778 ± 0.055, *p* = 0.9942; *CD74*: 0.863 ± 0.143, *p* = 0.2712; *CXCL-1*: 0.700 ± 0.122, *p* = 0.3389; *TIMP-1:* 0.963 ± 0.148, *p* = 0.7161; *TNF-α*: 1.453 ± 0.402, *p* = 0.6045; Figure 5A–C).

As shown before, crossbreeding of AROM**^+^** mice with mice lacking estrogen receptor α recovered the normal histological appearance of testicular sections without hyperplastic cells in the interstitial space and normal spermatogenesis. In this study, the strong TRPV2-immunoreactive staining in the interstitial space localized to the cellular membrane in 9 month-old AROM**^+^** animals (Figure 5D-D1), turned to only a weak and punctuated staining in the interstitial space of AROM^+^ mice crossed with ERαKO mice (Figure 5D-D3), similar to that observed in ERαKO animals (Figure 5D-D2).

### 2.6. TRPV2 in Adrenals and Brain

When we explored mRNA levels of *TRPV2* in the adrenal, another steroid-producing organ, from young and old WT and AROM^+^ animals (*n* = 3, each), we found that its levels slightly but not significantly increase with age in both WT (2.597 ± 0.618, *p* = 0.0613) and in AROM^+^ mice (2.997 ± 1.014, *p* = 0.1203) compared to the corresponding coeval animals. Expression levels of *TRPV2* in the immune-privileged organ brain, while readily detectable, did not change with age, neither in WT (1.210 ± 0.079, *p* = 0.0573), nor in AROM^+^ animals (1.277 ± 0.150, *p* = 0.1365). Taken together, the significantly increased expression levels of TRPV2 in the testis of old AROM^+^ mice (3.141 ± 0.538, *p* = 0.0163) compared to young ones, in contrast to WT animals showing no changes (1.310 ± 0.241, *p* = 0.2687), further confirm the testis-specificity of this observation (Figure S5).

## 3. Discussion

This study describes, for the first time, the expression pattern of the mysterious cation channel TRPV2 in murine testis with special regard to macrophages in the case of WT and the well-established model for male infertility AROM^+^, taking into account age-related changes. The results indicate that although TRPV2 is expressed by testicular macrophages in the WT mouse testis, expression levels are low and hence this channel likely is not to be considered a major factor involved in the regulation of testicular function during adult life and aging. However, the results of the analysis of testes from AROM^+^ animals imply that the population of TRPV2^+^ testicular macrophages massively increases with age induced by a misbalance of estrogen/androgen levels. It is likely that the testicular TRPV2^+^ macrophages are part of an inflammatory cascade in the testes of AROM^+^ and thereby are involved in the development of infertility.

TRPV2 could be detected and localized to interstitial cells of WT mice. However, the levels of TRPV2 were low, and the nature of the positive cells could not be readily identified by in situ hybridization studies. However, FACS isolation and subsequent RT-PCR showed that the TRPV2^+^ cells are mainly interstitial CD206^+^ macrophages rather than peritubular MHC II^+^ macrophages.

Pilot studies, namely the evaluation of organotypic incubations of mouse testes, indicated that in the presence of CB1 and CB2 blockers activation of testicular TRPV2 by CBD caused a detectable pro-inflammatory response, e.g., elevation of CXCL-1. The lack of more specific pharmacological tools, which would allow one to activate or block TRPV2 selectively, prevented us from further studies.

The magnitudes of the expression levels of TRPV2 and all analyzed macrophage or inflammation markers were not statistically significantly different in older WT animals. Of note, expression levels of the superoxide-producing enzyme *NOX2* increased, albeit only numerically with age. It may be a source of ROS, which have recently been described as natural activators and sensitizers of TRPV2 [28]. The role of NOX2 (and NOX1) was also reported to be critical for the differentiation of monocytes to macrophages and the polarization of M2 type but not M1 type macrophages [43]. In immune cells, including macrophages [44], such a mechanism of activation is very likely responsible for the regulation of macrophage functionality. Thus, the macrophage number may increase in the aged rodent testis [17], and their phenotype may undergo alteration, yet an active role of TRPV2 in the testes of old mice requires further investigation.

AROM^+^ testes are characterized, amongst other changes, by increased numbers of macrophages [1]. They have not yet been fully examined but the expression of CD68 and high TNF-α implicate a polarization towards M1 macrophages [15]. The high phagocytotic activity can be concluded from the observation that the macrophages engulf other cells, including Leydig cells [42]. We extended previous studies on this topic. We found that TRPV2 was readily detected in testes of AROM^+^ mice already at young adult ages. A FACS analysis performed with young mice revealed the unique nature of these cells. They constitute a group of CD206^+^MHC II^+^ cells, which is almost absent in normal WT mouse testis. Hence, the hormonal imbalances in the testes of these mice likely had initiated changes in the macrophage populations, resulting in the acquisition of this unique population. Whether the changes involve recruitment of a new macrophage population, as recently suggested to occur after radiation or infection [19] and whether or to which degree the factors of the local environment contribute to these changes [15], remain to be investigated.

The expression of TRPV2 and *NOX2* in the AROM^+^ testes further increased with age. As mentioned, NOX2 is reported to be critical for the differentiation of monocytes to macrophages [43], but it is also a source of ROS and could thereby activate and sensitize TRPV2. The analysis of expression profile of AROM^+^ testes gave hints of both, higher *NOX2* expression levels and more pro-inflammatory macrophages (evidenced, e.g., by higher inflammatory factors). The FACS analysis could not be readily performed in older animals, due to the altered and overall remodeled structure of the testes. Still, the evaluation of proteomic data of old animals indicated that several other macrophage markers increase in AROM^+^ animals (i.e., CAPG, CD68, CD206, MPEG1, and SIGLEC1). Furthermore, TRPV2 and NOX2 were exclusively found in AROM^+^ animals by this experimental approach, arguing against a role in normal aging in WT (see above) and for a role in AROM^+^.

Changes in testicular morphology and macrophage accumulation are initiated by hormonal imbalances and estrogen receptor α action, although the exact chain of events is unknown. It is assumed that increased estradiol levels stimulate Leydig cells to produce growth arrest-specific 6 (GAS6), which mediates phagocytosis of apoptotic cells by bridging cells with surface-exposed phosphatidylserine (PS) to macrophage receptors [45]. In general agreement with this assumption, we found that both the aromatase inhibitor treatment, as well as crossings of AROM^+^ with ERαKO, not only, as expected, rescued the infertile phenotype [6,42] and reverted macrophage and inflammation markers, but also reduced *TRPV2* and *NOX2* levels.

AROM^+^ mice have been studied for several years and changes in prostate, spleen, and testes were described. Higher plasma immunoglobulin levels in AROM^+^ mice are due to distinct changes in the spleen [4] and may indicate a generally altered immunological situation. Prostatitis and prostatic pre-malignancy occur in an age-dependent manner. Next to F4/80^+^ macrophages several other immune cell types were described in prostate. Increased macrophage numbers were first observed in the prostate of AROM^+^ mice at the age of 30 weeks and then increased with the advanced lifespan. In comparison, in AROM^+^ animals, testicular macrophages and testicular alterations are detectable at about 2 months of age, i.e., much earlier. Together with the other changes mentioned, this suggests organ-specific changes. We further monitored *TRPV2* levels in adrenals and brains of male AROM^+^ and WT animals (see Supplementary Materials, Figure S5). We chose the brain since similar to the testis, it is an immune-privileged organ. However, we did not find any changes in *TRPV2* expression in AROM^+^ compared to WT and no evidence for age-associated alterations either. In contrast, in steroid-producing adrenals, *TRPV2* increased with age but in a similar fashion in both, WT and AROM^+^ mice. The results indicate that in the adrenal, which is similar to the testis produces steroids, age is the major factor in the induction of *TRPV2*. The results require additional studies, but taken together, the distinct changes in the mentioned organs indicate that changes in TRPV2 and macrophages occur in a highly organ-specific way.

In summary, our study further defines the phenotypes of testicular macrophages in the mouse. TRPV2^+^ macrophages normally reside in the mouse testis, yet are of limited number and do not change significantly with age. Hormonal imbalances, however, initiate a chain of events, in which the number of TRPV2^+^ macrophages increases. This situation causes the appearance of a CD206^+^MHC II^+^ subpopulation of TRPV2^+^ macrophages, which appear to be part of the inflammatory events in AROM^+^ animals and may play roles in testicular dysfunction and infertility.

## 4. Materials and Methods

### 4.1. Animals

For this study, animals of the well-established mouse model AROM^+^ (human P450arom transgene expressed under the ubiquitin C promotor) with the corresponding WT littermates were used and age-grouped for different approaches. Moreover, 1 month-old WT and AROM**^+^** animals either treated with placebo (5 mg/mL carboxylmethyl-cellulose in deionized water, Tamro Ltd., Vantaa, Finland) or 10 mg/kg aromatase inhibitor, finrozole (MPV-2213ad, Hormos Medical Ltd., Turku, Finland) for 6 weeks were included into this study. Further, estrogen receptor α knock-out mice, ERαKO, and ERαKO crossbred with AROM**^+^** animals were histologically investigated. In addition to the phenotypic control (cryptorchidism, smaller and brownish testes), the expression of the hP450 transgene (*hCYP19A1*) in AROM^+^ mice was verified by means of PCR. The generation and treatment of these genetically modified mice has been described in detail elsewhere [1,2,6,7,41,42,46]. The animals had free access to soy-free food pellets (SDS; Witham, Essex, England) and tap water and were handled in accordance with the institutional animal care policies of the University of Turku (Turku, Finland).

### 4.2. Immunohistochemistry and In Situ Hybridization of Testicular Sections

After antigen retrieval using 10 mM citrate buffer at pH 6, sections from Bouin’s solution-fixed and paraffin embedded mouse testicular samples of all the above-mentioned genotypes—AROM**^+^**, ERαKO, crossbreeding of these and corresponding WT animals, respectively—were immunohistochemically investigated for the presence of TRPV2 using a polyclonal antibody produced in rabbit (1: 400; HPA044993; Sigma-Aldrich, St. Louis, MO, USA). Additionally, consecutive testicular sections of AROM**^+^** and the corresponding WT animals were scanned for the murine macrophage marker F4/80 (monoclonal antibody produced in rat; 1: 50; MCA497; AbD Serotec, Puchheim, Germany). Accordingly, biotinylated goat-α-rabbit (1: 2500; BA-1000; Vector Laboratories, Inc., Burlingame, CA, USA) and goat-α-rat (1: 1000; AB_2338179; Jackson Immuno Research Laboratories, Inc., Ely, Cambridgeshire, UK) secondary antibodies were used, followed by application of an avidin-biotin-complex peroxidase (ABC, Vector Laboratories, Inc. Burlingame, CA, USA) and DAB (Sigma-Aldrich, St. Louis, MO, USA). Primary antibodies omitted, non-immune serum or pre-adsorbed antibody (1: 100; APrEST83822, Sigma-Aldrich, St. Louis, MO, USA) served as negative controls. *TRPV2* in situ hybridization was performed on PFA-fixed (10%) and paraffin embedded testicular sections from 2 and 10 month-old AROM**^+^** and corresponding WT littermates using the RNAscope 2.5 HD-Brown Assay (ACD, Inc., Newark, NJ, USA) with probes against murine *TRPV2* (Cat No. 522811), bacterial *DapB* as negative control [(−) ctrl.] and *PPIB* as positive control [(+) ctrl.] in accordance to the user manual. Sections from both immunohistochemistry and in situ hybridization were slightly counterstained with hematoxylin.

### 4.3. Western Blot

Protein was isolated from bisected testes of young (3 months, *n* = 2) and old AROM**^+^** animals (7 months, *n* = 2) and the corresponding WT littermates (*n* = 2, each) by disruption of the tissue using lysing tubes containing ceramic beads (Lying Matrix D; MP BiomedicalsTM; Irvine, CA, USA) and RIPA buffer supplemented with protease and phosphatase inhibitors (A32959; Thermo Fisher Scientific Inc., Waltham, MA, USA) in adequate volumes in a tissue homogenizer (FastPrep-24TM 5G; MP BiomedicalsTM; Irvine, CA, USA) with 2–3 cycles at 6.0 m/s speed for 120 s with pauses of 2 min on ice, followed by sonification and centrifugation at 13,000 rpm for 15 min. The sample concentration was measured by Lowry assay (DCTM Protein Assay; Bio-Rad Laboratories, Inc., Hercules, CA, USA), and equal amounts of protein (10 µg/lane) were loaded. The membrane was incubated with a polyclonal TRPV2 antibody (1: 600; HPA044993; Atlas Antibodies, Stockholm, Sweden) at 4 °C over-night and a monoclonal αTubulin antibody (1: 10,000; ab52866; Abcam, Cambridge, UK) for 1 h at room temperature, both produced in rabbit. An IRDye 800CW secondary antibody donkey-α-rabbit (1: 10,000; 926-32213; Li-COR Biosciences, Lincoln, NE, USA) was used and bands were detected with the Odyssey CLx imaging system (Li-COR Biosciences, Lincoln, NE, USA). Intensities were measured, background subtracted and normalized to αTubulin.

### 4.4. Quantitative RT-PCR

Disruption of testicular, adrenal, and brain tissue, followed by isolation of mRNA, was done using the RNeasy Plus Universal Mini Kit (Qiagen, Hilden, Germany) following the manufacturer’s protocol. The quantity of mRNA was measured using a spectrophotometrical approach (NanoDrop 2000c, Thermo Fisher Scientific Inc.) and the purity of isolated RNA was monitored by the 260/280 ratio. After that, mRNA was subjected to reverse transcription (SuperScriptTM II Reverse Transcriptase; Invitrogen, Carlsbad, CA, USA). Then, 4–10 ng cDNA were used for quantitative RT-PCR (LightCycler 96^®^ System, Roche Diagnostics, Penzberg, Germany) using the QuantiFast SYBR Green PCR kit (Qiagen, Hilden, Germany). Ribosomal protein L19 (L19) served as an internal control and was used for normalization. The statistical analysis was performed on the ΔCq values of each animal of treatment (for further details, see Section 4.9) and data are presented as changes in gene expression relative to the corresponding control group according to the 2^−∆∆Cq^ method, as described elsewhere [47]. PCR products were loaded on a 2% agarose gel supplemented with Midori Green Advance DNA stain (Nippon Genetics Europe, Düren, Germany) for visualization, then separated, eluted, and sent for sequencing to verify their identity (GATC, Konstanz, Germany). Nucleotide sequences of the utilized primers and the size of the corresponding PCR products are given in Table 1.

### 4.5. Mass Spectrometry

#### 4.5.1. Sample Preparation

Approximately 1 mg of tissue was incised from 11 month-old WT and AROM^+^ mice testis (*n* = 3, each). The tissues were homogenized manually using Micro-homogenizers, PP (Carl Roth GmbH+Co. KG, Karlsruhe, Germany) and proteins were extracted using the iST Sample Preparation Kit (PreOmics, Martinsried, Germany) using the manufacturer’s protocol. Briefly, tissues were lysed and subsequently sheared to get rid of DNA and other interfering molecules followed by incubation with Trypsin at 37 °C for 2.5 h. The resulting peptides were subsequently desalted, purified, and re-dissolved in 10 µL ‘Load’ solution after drying completely in a speed vac.

#### 4.5.2. Mass Spectrometry Measurements

Reversed phase HPLC separation of peptides was performed on an Ultimate 3000 nanoLC system (Thermo Fisher Scientific Inc., Waltham, MA, USA). In addition, 5 μL of the solution was loaded onto the analytical column (120 × 0.075 mm, in the house packed with ReprosilC18-AQ, 2.4 μm, Dr. Maisch GmbH, Ammerbuch-Entringen, Germany), washed for 5 min with 3% ACN containing 0.1% FA at 300 nl/min, and subsequently separated applying a linear gradient from 3% ACN to 40% ACN over 50 min. Peptides eluted were ionized in a nanoESI source and on line detected on a QExactive HF mass spectrometer (Thermo Fisher Scientific Inc., Waltham, MA, USA). The instrument was operated in a TOP10 method in a positive ionization mode, detecting eluting peptide ions in the *m*/*z* range from 375 to 1600 and performing MS/MS analysis of up to 10 precursor ions. Peptide ion masses were acquired at a resolution of 60,000 (at 200 *m*/*z*). High-energy collision-induced dissociation (HCD) MS/MS spectra were acquired at a resolution of 15,000 (at 200 *m*/*z*). All mass spectra were internally calibrated to lock masses from ambient siloxanes. Precursors were selected based on their intensity from all signals with a charge state from +2 to +5, isolated in a 2 *m*/*z* window and fragmented using a normalized collision energy of 27%. To prevent repeated fragmentation of the same peptide ion, the dynamic exclusion was set to 20 s.

#### 4.5.3. Data Analysis

Protein identification was performed by the MaxQuant 1.6.0.16 software package. Parent ion and fragment mass tolerances were 8 ppm and 0.7 Da, respectively and two missed cleavages were allowed. The mouse canonical protein database from Uniprot (release June, 2018), filtered to retain only the reviewed entries was used for the searches. Regular MaxQuant conditions were the following: Peptide FDR, 0.01; Protein FDR, 0.01; Min. peptide Length, 5; Variable modifications, Oxidation (M); Acetyl (Protein N-term); Acetyl (K); Dimethyl (KR); Fixed modifications, Carbamidomethyl (C); Peptides for protein quantitation, razor and unique; Min. peptides, 2; Min. ratio count, 2. Proteins were validated on the basis of at least 1 unique peptide detected.

### 4.6. Cell Sorting with Flow Cytometry

Mice were euthanized at the age of 2 or 7–8 months by CO_2_ asphyxiation following cervical dislocation or cardiac puncture. Testes were removed, minced and digested with 50 µg/mL DNase 1 and 1 mg/mL Collagenase D in Hanks’ solution at 37 °C for 45 min. The isolated cells were washed and suspended in PBS, and the cell suspension was filtered through a silk cloth (pore size 77 µm). Cells were incubated with unconjugated CD16/32 antibody (clone 2.4G2; Bio X Cell, Lebanon, NH, USA) to block the unspecific binding to low-affinity Fc-receptors and then stained for 30 min at 4 °C with the following antibodies: Anti-CD45-PerCP-Cy5,5 (clone 3O-F11; BD, Franklin Lakes, NJ, USA), anti-F4/80-A488 (clone BM8; eBioscience, San Diego, CA, USA), anti-CD11b-BV786 (clone M1/70; BD, Franklin Lakes, NJ, USA), anti-CD206-BV650 (clone C068C2; BioLegend, San Diego, CA, USA), anti-MHC II-PE (clone M5/114.15.2; BD, Franklin Lakes, NJ, USA), anti-CD11c-BV711 (clone HL3; BD, Franklin Lakes, NJ, USA), anti-Ly6C-BV421 (clone AL-21; BD, Franklin Lakes, NJ, USA), anti-TRPV2 (HPA044993; Sigma-Aldrich, St. Louis, MO, USA) conjugated with a secondary α-Rb A647 (A27040, Invitrogen, Carlsbad, CA, USA), diluted to the FACS-buffer. Cells were washed and fixed with 1% formaldehyde in PBS. Samples were acquired with the LSRFortessa flow cytometer (BD, Franklin Lakes, NJ, USA), and data were analyzed with the FlowJo software (FlowJo LLC, Ashland, OR, USA).

For subsequent quantitative RT-PCR analysis, wild-type CD45**^+^**CD11b**^+^**F4/80**^+^** cells were sorted for either CD206**^+^**MHC II**^-^** or CD206**^-^**MHC II**^+^** using a Sony SH800 cell sorter (100 µm nozzle, Sony Biotechnology Inc., San Jose, CA, USA). The purity of the isolated populations was >95%. The gating strategies for flow cytometric analyses are shown in Figure S4.

### 4.7. Organotypic Testicular Tissue Incubation

Freshly isolated testes from young WT animals (2–3 months, *n* = 7) were decapsulated, divided into four equally sized tissue pieces, placed into individual wells of a 24-well plate containing DMEM/F12 (Gibco, Paisley, UK) and incubated with CB1 (AM251, 80 nM; Tocris Bioscience, Bristol, UK) and CB2 blockers (AM630, 800 nM; Tocris Bioscience, Bristol, UK), respectively, for 1 h at 37 °C, 5% CO_2_, and 95% humidity on a rocking platform. After this pre-incubation, CBD (Tocris Bioscience, Bristol, UK) was added at a final concentration of 30 µM, with EtOH in equal volume as the solvent control and the tissue pieces were incubated for another 6 h. Afterwards, tissue pieces were frozen in liquid nitrogen, stored at −80 °C for RNA isolation. Supernatants were collected, frozen, and stored for further analysis in a proteome profiler.

### 4.8. Proteome Profiler

For this Proteome Profiler Mouse Cytokine Array Panel A (R&D Systems, Minneapolis, MN, USA) supernatants of incubated testicular tissues treated with 30 µM CBD or the EtOH solvent control of one 2.5 month-old wild-type animal were used following the manufacturer’s instructions. Briefly, membranes were blocked and incubated with a mixture consisting of supernatant and corresponding buffer overnight. On the next day, the membrane was incubated with Detection Antibody Cocktail followed by streptavidin-horseradish-peroxidase for 1 h each. Finally, a Chemi Reagent Mix was added to measure chemiluminescence with an exposure time of 5 min. The average spot signal density was measured, background subtracted, and normalized to the mRNA amount.

### 4.9. Data Analysis and Statistics

The testicular section of the above-listed animals subjected either to *TRPV2* in situ hybridization or immunohistochemistry against F4/80 and TRPV2 were visualized using a Zeiss Axioplan microscope equipped with a Plan-Neofluar 40x/0.75 objective (Carl Zeiss Microscopy, Jena, Germany) and a Jenoptik camera (PROGRES GRYPHAX Arktur; Jenoptik, Jena, Germany) with the corresponding software (PROGRES GRYPHAX^®^, Version 1.1.8.159 for Macintosh, 2017, Jenoptik Optical Systems GmbH, Jena, Germany). Microscopic images were further white-balanced and brightness was adjusted using Fiji (open source image processing package for ImageJ).

Quantitative RT-PCR datasets were analyzed using Microsoft Excel (2018, Microsoft, Redmond, WA, USA) and the statistical analysis was performed on the ΔCq values of each animal/treated tissue using Prism 7 (GraphPad, San Diego, CA, USA). After outlier exclusion (ROUT method, Q = 1%), datasets were checked for Gaussian distribution using the Shapiro-Wilk normality test (α = 0.05) and were subjected either to an unpaired (animals with different age, genotype and treatment, unpaired conditions) or paired (organotypic testicular tissue incubation, paired conditions) two-tailed t-tests. Datasets of wild-type macrophages sorted for their CD206 and MHC II expression and further subjected to quantitative RT-PCR looking for *TRPV2* (Figure 1F) were not statistically investigated due to the low *n* in this approach (2 months, *n* = 1, pool of three animals; 7–8 months, *n* = 2, pool of three animals each).

For analysis of mass spectrometry datasets, the measured iBAQ intensities were checked for Gaussian distribution using the Shapiro-Wilk normality test (α = 0.05) followed by an unpaired two-tailed *t*-test, for those proteins detected in all three biological replicates of each group (3/3). The differential protein expression between the two genotypes is expressed as fold change based on the mean WT log2 iBAQ intensity.

For analysis of the FACS datasets, calculated percentages of the corresponding macrophage subpopulations were checked for Gaussian distribution using the Shapiro-Wilk normality test (α = 0.05), and were further subjected to an unpaired two-tailed *t*-test when all three biological replicates of each group (3/3) showed values greater than 0.

For all the tests, α was set to 0.05 (* *p* ≤ 0.05, ** *p* ≤ 0.01, *** *p* ≤ 0.001), and data are depicted as mean ± SEM.

Signal densities of Western Blot bands and Proteome Profiler spots were measured using Fiji and further quantifications were done with Excel and Prism 7. Due to the low *n* in both approaches (Western Blots: *n* = 2 for each group; proteome profiler: *n* = 1), no statistical analysis was performed.

## Figures and Tables

**Table 1:** Oligonucleotide primer sequences and corresponding amplicon sizes.

Gene	Reference ID	Nucleotide Sequence	Amplicon Size (bp)
*hCYP19A1*	NM_000103	5′-GCT ACC CAG TGA AAA AGG GGA-3′ 5′-GCC AAA TGG CTG AAA GTA CCT AT-3′	140
*L19*	NM_009078.2	5′-AGG CAT ATG GGC ATA GGG AA-3′ 5′-CC ATG AGG ATG CGC TTG TTT-3′	199
*CD54*	NM_010493.3	5′-TGG AGA CGC AGA GGA CCT TA-3′ 5′-CAG TGT GAA TTG GAC CTG CG-3′	182
*CD68*	NM_009853.1	5′-CCA GCT GTT CAC CTT GAC CT-3′ 5′-AGA GGG GCT GGT AGG TTG AT-3′	208
*CD74*	NM_001042605.1	5′-GCC ACC ACT GCT TAC TTC CT-3′ 5′-GTT CTT CAC AGG CCC AAG GA-3′	198
*CD206*	NM_008625.2	5′-GAG CCC ACA ACA ACT CCT GA-3′ 5′-TCG CCA GCT CTC CAC CTA TA-3′	157
*COX2*	NM_011198.4	5′-CTT CGG GAG CAC AAC AGA GT-3′ 5′-TTC AGA GGC AAT GCG GTT CT-3′	225
*CXCL-1*	NM_008176.3	5′-AGT TCC AGC ACT CCA GAC TC-3′ 5′-AGT GTG GCT ATG ACT TCG GT-3′	246
*CXCL-2*	NM_009140.2	5′- TCA ATG CCT GAA GAC CCT GC-3′ 5′- TTT GAC CGC CCT TGA GAG TG-3′	119
*IL-1ß*	NM_008361.3	5′-TGA AGT TGA CGG ACC CCA AA-3′ 5′-TGA TGT GCT GCT GCG AGA TT-3′	101
*IL-6*	NM_031168.2	5′-TTG GGA CTG ATG CTG GTG AC-3′ 5′-CAG GTC TGT TGG GAG TGG TAT-3′	91
*MCP-1*	NM_011333.3	5′-GGC TCA GCC AGA TGC AGT TAA-3′ 5′-CCA GCC TAC TCA TTG GGA TCA-3′	80
*TIMP-1*	NM_001044384.1	5′-TTC TTG GTT CCC TGG CGT AC-3′ 5′-GCA AAG TGA CGG CTC TGG TA-3′	191
*TNF-α*	NM_013693.3	5′-CAC AGA AAG CAT GAT CCG CG-3′ 5′-TGA TGA GAG GGA GGC CAT TTG-3′	209
*TRPV2*	NM_011706.2	5′-CGA TGA GTT CTA CCG AGG CC-3′ 5′-TCA CCA CAT CCC ACT GCT TG-3′	206
*NOX2*	NM_007807.5	5′-GAG GTT GGT TCG GTT TTG GC-3′ 5′-CAG GAG CAG AGG TCA GTG TG-3′	191

## Data Availability

Not applicable.

## Supplementary Materials

The following are available online at https://www.mdpi.com/article/10.3390/ijms22094727/s1.
