# Supplementary material for: Exploring the Ion Channel TRPV2 and Testicular Macrophages in Mouse Testis
Source: Int J Mol Sci. 2021 Apr 29;22(9):4727. doi: 10.3390/ijms22094727 (PMC8124949; doi:10.3390/ijms22094727)
Supplement: Supplementary file 1 [file ijms-22-04727-s001.zip › ijms-22-04727-s001.pdf]

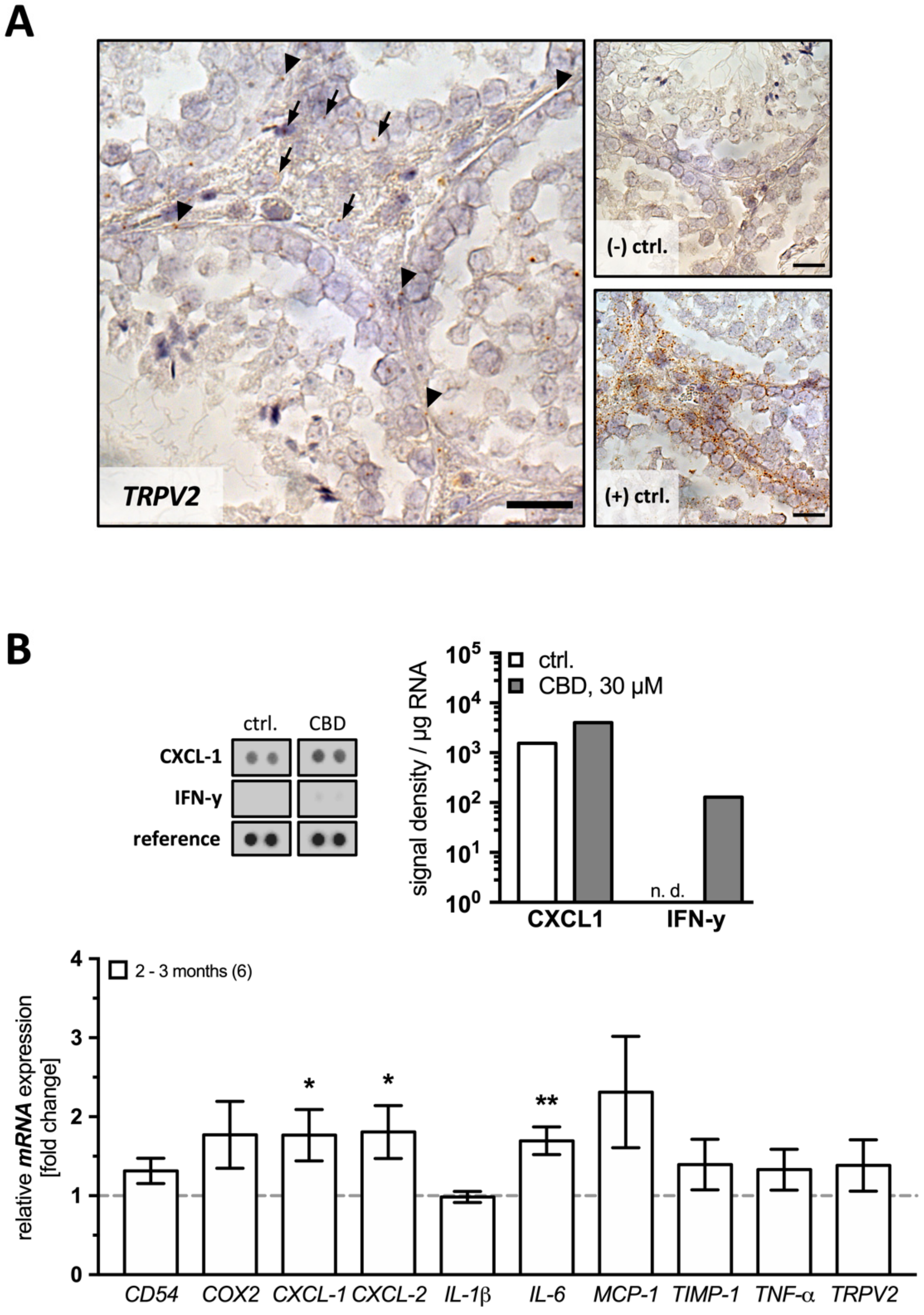

Supplementary Figure S1. TRPV2 in testicular sections from young and old WT mice and effects of CBD in testicular tissue incubation experiments. (**A**) 2 months-old WT mice subjected to *TRPV2* in situ hybridization (left panel) revealed punctuated staining both in the interstitial space (arrows) and within or in close proximity to the peritubular wall (arrow heads). Negative control (upper right panel, (-) ctrl.) showed no signals, but the positive control (lower right panel, (+) ctrl.) did. Scale bar 50 µm. (**B**) Testicular tissue from young WT mice incubated with 30 µM CBD together with CB1 and 2 blockers (AM251 and AM630, respectively) for 6 h showed higher CXCL-1 and IFN-y levels in the supernatant, analyzed by a cytokine profiler (upper panel). mRNA levels of *CXCL-1* (1.767 ± 0.326; p = 0.0493) and *2* (1.807 ± 0.334; p = 0.0018), as well as *IL-6* (1.697 ± 0.174; p = 0.0066) increased significantly, whereas *CD54* (1.315 ± 0.159; p = 0.0968), *COX2* (1.772 ± 0.425; p = 0.0919), *IL-1ß* (0.985 ± 0.070; p = 0.7045), *MCP-1* (2.313 ± 0.705; p = 0.0527), *TIMP-1* (1.395 ± 0.320; p = 0.4632), *TNF-α* (1.330 ± 0.258; p = 0.3588) and *TRPV2* (1.383 ± 0.325; p = 0.4549) remained unchanged at the same time (n = 6). Graphs represent mean ± SEM compared to solvent (EtOH) treated testicular tissue; paired two-tailed t-test, $\alpha = 0.05$.

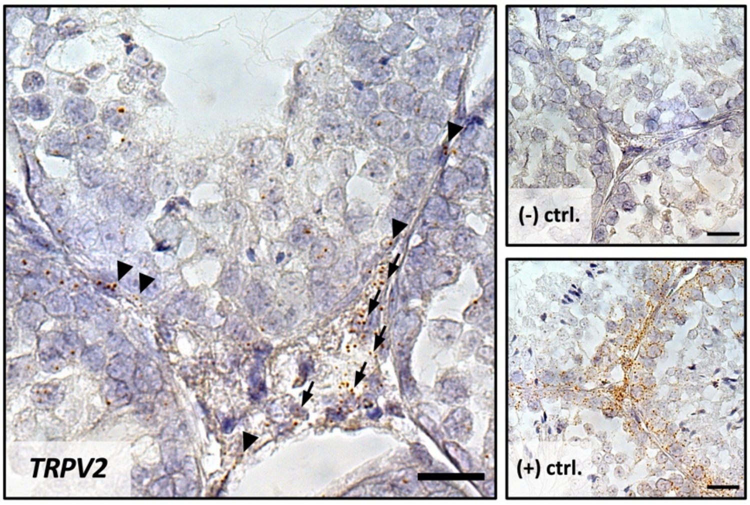

Supplementary Figure S2. ***TRPV2*** **in situ hybridization of young, 2 months-old AROM⁺ mice.** Testicular sections of 2 months-old AROM⁺ mice subjected to *TRPV2* in situ hybridization (left panel) revealed strong and punctuated staining mainly in the interstitial space (arrows), but also revealed TRPV2 signals within or in close proximity to the peritubular wall (arrow heads). Negative control (upper right panel, (-) ctrl.) showed no signals, but the positive control (lower right panel, (+) ctrl.) did. Scale bar 50 µm.

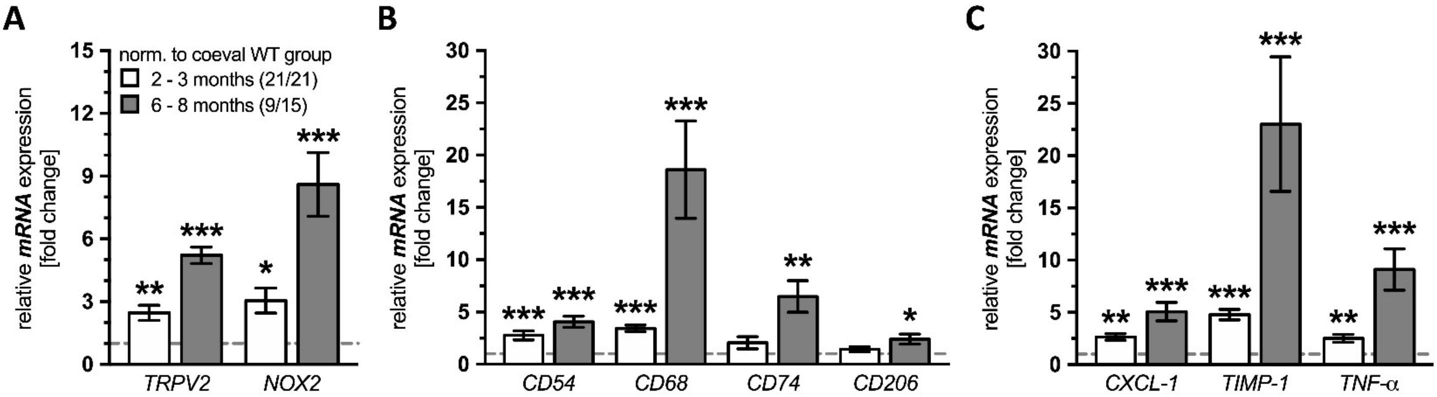

Supplementary Figure S3. Genotypic comparison of expression profiles in young and old WT and AROM⁺ testes. (**A-C**) Genotype-dependent comparison of mRNA expression levels of (**A**) *TRPV2* and *NOX2*, (**B**) macrophage and (**C**) inflammation markers in whole testis from young and old AROM⁺ mice (n = 9 and 15, respectively) compared to coeval WT animals (n = 21, each). mRNA expression levels were always significantly higher in AROM⁺ animals, except for *CD74* (2.061 ± 0.582, p = 0.7800) and *CD206* (1.431 ± 0.229; p = 0.4744) in the young animals. Graphs represent mean ± SEM; unpaired two-tailed t-test, $\alpha = 0.05$.

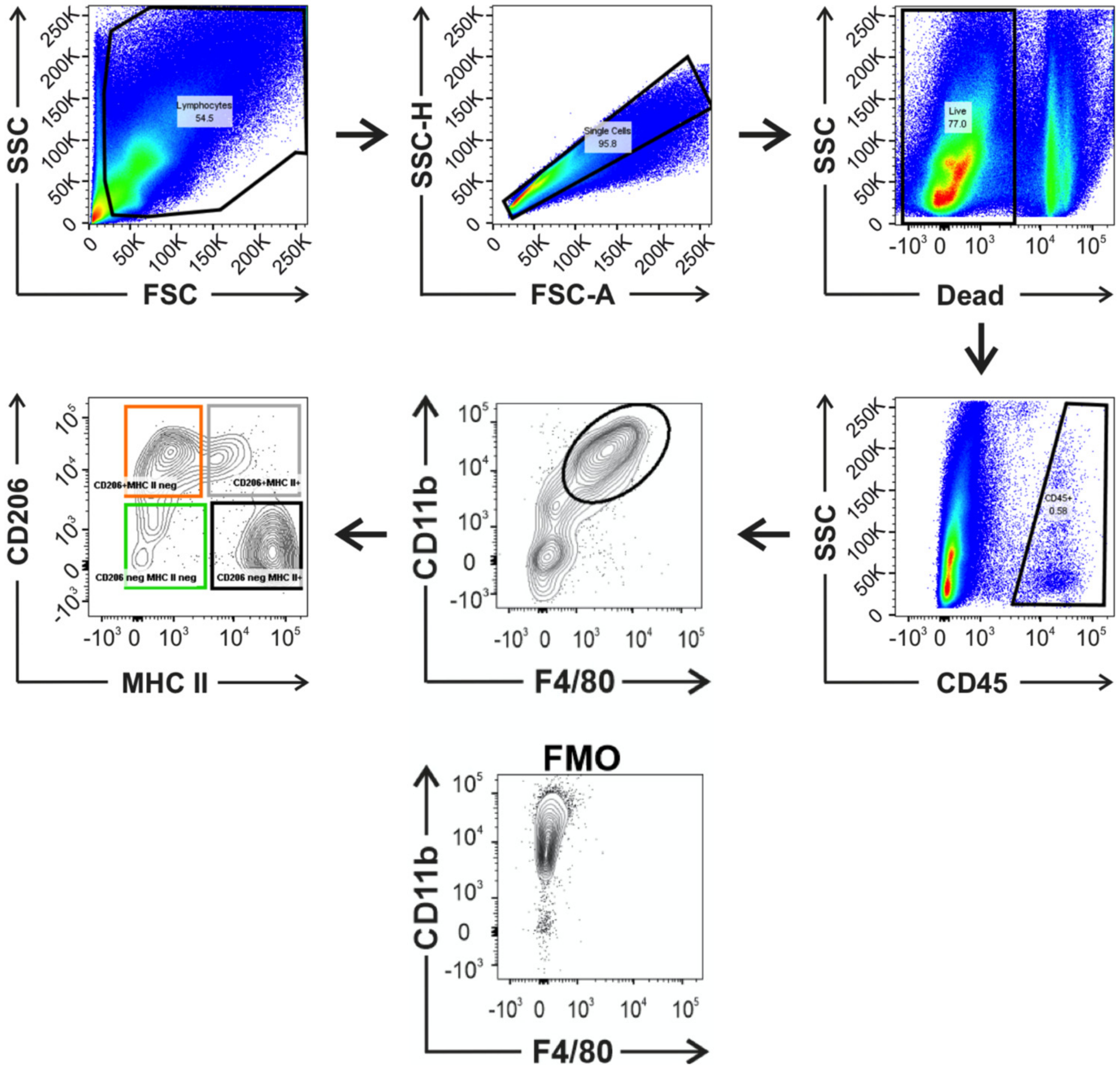

Supplementary Figure S4. Gating strategies for flow cytometry of testicular macrophages. FACS gating strategy with representative two-dimensional plots for murine testicular macrophages and fluorescence minus one (FMO) control. Living cells were gated for $CD45^+$ $F4/80^+CD11b^+$ events before downstream analysis and were further sorted for MHC II and CD206, resulting in four macrophage subpopulations: $CD206^-$MHC II^- (green gate), $CD206^+$MHC II^- (orange gate), $CD206^-$MHC II^+ (black gate) and $CD206^+$MHC II^+ (gray gate).

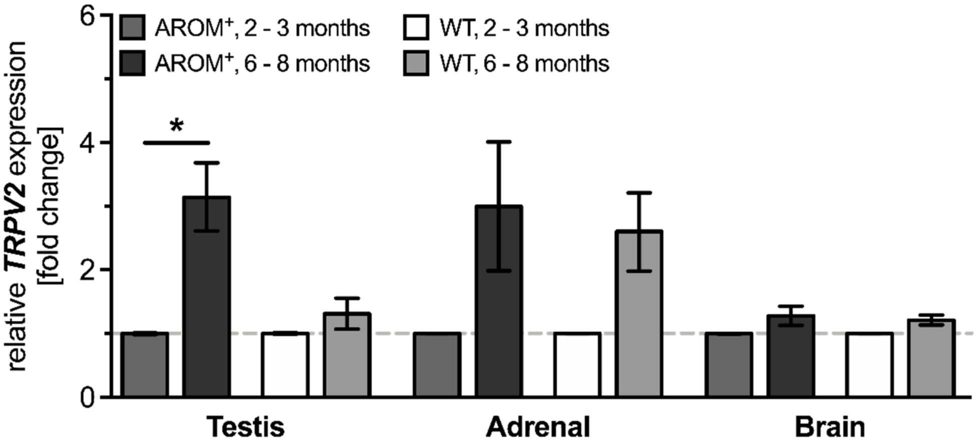

Supplementary Figure S5. ***TRPV2*** **expression in testis, adrenal and brain of young and old WT and AROM+ mice.** mRNA extracted from testis, adrenal and brain of young (2 - 3 months) and old (6 - 8 months) WT and AROM+ mice (n = 3, each) was subjected to quantitative RT-PCR revealing significantly higher *TRPV2* expression levels in testicular tissue from old AROM+ animals compared to the young ones (3.141 ± 0.538, $p = 0.0163$), but not in tissue from WT mice (1.310 ± 0.241, $p = 0.2687$). *TRPV2* expression levels were also numerically elevated in adrenal from old WT (2.597 ± 0.618, $p = 0.0610$) and AROM+ (2.997 ± 1.014, $p = 0.1203$) compared to the corresponding young animals, but did not reach significance. In brain, also immune privileged, *TRPV2* expression was unchanged (WT: 1.210 ± 0.079, $p = 0.0573$; AROM+: 1.277 ± 0.150, $p = 0.1365$). Graphs represent mean ± SEM; unpaired two-tailed t-test, $\alpha = 0.05$.
